# Practicing Other-Focused Kindness and Self-Focused Kindness Among Those at Risk for Mental Illness: Results of a Randomized Controlled Trial

**DOI:** 10.3389/fpsyg.2021.741546

**Published:** 2021-10-14

**Authors:** S. Katherine Nelson-Coffey, Ernst T. Bohlmeijer, Marijke Schotanus-Dijkstra

**Affiliations:** ^1^Department of Psychology, The University of the South, Sewanee, TN, United States; ^2^Department of Psychology, Health and Technology, Centre for eHealth and Well-Being Research, University of Twente, Enschede, Netherlands

**Keywords:** kindness, prosocial behavior, self-focused behavior, well-being, positive psychology intervention, flourishing, anxiety, depression

## Abstract

People with reduced levels of mental well-being might be at risk for developing future mental illness. Although several positive psychology interventions successfully improve mental well-being and psychological distress, less is known about their efficacy in a sample at risk for mental disorders. A Dutch sample of 289 participants with low or moderate levels of well-being were randomly assigned to other-focused kindness with reflection, other-focused kindness without reflection, self-focused kindness, or waitlist control (Trial register: NTR6786). Results of multilevel growth curve analyses revealed that other-focused kindness, but not self-focused kindness, led to improvements in the primary outcome mental well-being relative to waitlist control up to 6-week follow-up. By contrast, only other-focused kindness without reflection led to improvements in psychological distress. The three kindness conditions mainly did not differ from one another, and mainly no differences were found up to 6-months follow-up. An exception was that perceived stress was significantly more reduced up to 6-week and 6-months follow-up when people practiced other-focused kindness without reflection then when participants had practiced self-focused kindness. These findings point to the benefits of practicing kindness for others when people might be at risk for future mental illness. The study also indicates that reflecting about practicing kindness does not seem to have added value.

## Introduction

Promoting mental well-being is now seen as an important direction for public mental health and clinical psychology (e.g., Keyes, 2007a; Wood and Tarrier, 2010; Kobau et al., 2011; Jeste et al., 2015; Huber et al., 2016). A potential new target group for positive mental health promotion consists of people who are not flourishing and have also no severe mental health complaints. For example, Keyes (2007b) has shown that 60.3% of the US population did not have a mental illness, but were also not able to flourish. Similarly, 53% of the people in the Netherlands reported reduced levels of mental well-being but were not diagnosed with a mental disorder (Schotanus-Dijkstra et al., 2016, 2017b). Recent evidence shows that people with low or moderate levels of well-being report infrequent positive emotions (Catalino and Fredrickson, 2011), less positive life-events (Schotanus-Dijkstra et al., 2016), and an increased risk of developing a diagnosed mood or anxiety disorder 3 years later (Schotanus-Dijkstra et al., 2017b). In addition, people who stay in the low or moderate range of well-being within a 10-year time span are three times more likely to have developed a mental disorder during that period than those who stayed flourishing (Keyes et al., 2010).

These and other findings indicate that flourishing mental health should be promoted in people with reduced levels of well-being (Keyes et al., 2010; Wood and Joseph, 2010; Grant et al., 2013; Lamers et al., 2015; Schotanus-Dijkstra et al., 2017b). Several meta-analyses have shown that mental well-being and psychological distress can be improved through a variety of interventions, such as gratitude interventions, kindness interventions, and self-compassion interventions (Sin and Lyubomirsky, 2009; Bolier et al., 2013; Galante et al., 2014; Weiss et al., 2016; Dickens, 2017; Curry et al., 2018; Hendriks et al., 2018, 2019). Yet, most studies have been conducted within student samples (e.g., Layous et al., 2013a), healthy populations (e.g., Fredrickson et al., 2008), or clinical populations (e.g., Chaves et al., 2016), and only a few specifically targeted those with low or moderate levels of well-being but without any clinical symptoms (e.g., Schotanus-Dijkstra et al., 2017a). Positive psychology interventions used for selective and indicated prevention purposes may be especially effective, as those people are likely motivated to improve their well-being (Lyubomirsky et al., 2011). People with low levels of well-being might also benefit more from positive psychology interventions than those with higher levels of well-being (Nelson et al., 2014). A good start to test the efficacy of positive interventions in those at risk for developing mental disorders, is by examining practicing kindness.

A great advantage of practicing kindness is that it is a simple intervention that could be easily integrated in daily life and clinical practice and may cost no money, although some studies specifically focus on prosocial spending of which its emotional benefits are well-established in the literature (Aknin et al., 2013, 2020). In the current study, the focus lies on performing acts of kindness in which participants are typically asked to perform nice things for others, such as picking up groceries for a sick neighbor or giving a compliment to a colleague. Performing acts of kindness has been reliably linked to improved well-being (see Curry et al., 2018 for a meta-analysis). However, Curry et al. (2018) noted in their review of the literature that the majority of kindness studies (including acts of kindness and prosocial spending) focus primarily on immediate benefits of kindness for subjective well-being. Yet, it remains unclear whether performing kindness is effective in improving overall mental well-being and psychological distress because most studies to date mainly focus on happiness, life satisfaction, and positive affect (e.g., Alden and Trew, 2013; Layous et al., 2013b; Curry et al., 2018), and the studies regarding psychological distress are inconclusive (Mongrain et al., 2011; Kerr et al., 2015). For example, practicing kindness significantly reduced stress and anxiety compared to keeping track of daily moods, but it had no effect on reducing depressive symptoms (Kerr et al., 2015). Another study also found no effect of practicing kindness on a daily basis for 1 week in comparison with an early-memory control condition (Mongrain et al., 2011).

### The Added Value of Reflection

To expand prior knowledge about the efficacy of kindness further, we also explored whether adding reflection to performing acts of kindness is more beneficial for mental health than performing acts of kindness without reflection. This hypothesis was based on prior studies suggesting that reliving a positive event might be even more important for feeling good than the activity itself (e.g., Emmons and McCullough, 2003; Van Boven and Gilovich, 2003). The emotional benefits of expressive writing is well-established in the literature, although the focus often lies in writing about traumatic or emotionally charged experiences (Pennebaker, 2018). However, when dating couples expressively wrote about their relationships, the chance of being together 3 months later was significantly higher compared to couples who wrote about daily activities (Slatcher and Pennebaker, 2006). In addition, a recent study demonstrated that *recalling* three kind acts on 1 day revealed similar improvements on different well-being outcomes (e.g., positive and negative affect, life-satisfaction) relative to *performing* three kind acts on 1 day or doing both (Ko et al., 2021). Although this was a 3-day trial, these findings might indicate that reflecting on past kind acts might be at least as effective as performing kind acts.

Furthermore, a study by Grant and Dutton (2012) demonstrated that participants who reflected about recent performed kind acts for others called more potential fundraisers and donated more money to charity compared to participants who reflected about receiving kind acts. However, a certain amount of engagement in a reflection task after performing kind acts seems a prerequisite for its beneficial effects (Aknin et al., 2020). For example, receiving instructions such as “describe your purchase as vividly and in as much detail as possible” (Aknin et al., 2020) was more generic which might have led to smaller effects than instructions such as “reflect on what brought it about, and describe how it affected your thoughts, feelings and actions” (Grant and Dutton, 2012). In the current study, we add specific reflection instructions about the kind acts they performed 1 day earlier and compared this with performing kind acts for others without reflection.

### Other-Focused Kindness vs. Self-Focused Kindness

Interestingly, Western cultures often emphasize taking care of oneself first before helping others. The popularity of self-compassion may have strengthened this vision, as self-compassion is associated with higher levels of mental well-being (Leary et al., 2007; Neff et al., 2007; Gilbert, 2010). Being kind to one self, especially in the face of suffering and failure, is one of the three aspects of self-compassion (Neff, 2003). However, this aspect differs from popular perceptions of self-kindness, which may be more commonly interpreted as self-indulgent behavior.

In fact, accumulating evidence shows that it might be more worthwhile for people's mental well-being to help others and build positive relationships (Dunn et al., 2011; Nelson et al., 2016; Crocker et al., 2017; Schotanus-Dijkstra et al., 2019). For instance, a recent experimental study found greater increases in positive affect for participants who recalled an act of kindness with the intention to gain benefits for the other person compared to participants who recalled an act of kindness with the intention to gain benefits for themselves (Wiwad and Aknin, 2017). In addition, kindness to benefit others was coded as being more kind and impactful compared to performing kind acts for others with a focus on the self (Wiwad and Aknin, 2017). Similarly, a study among adults recruited from the general US population showed that performing five acts of kindness for others on 1 day was significantly more effective in enhancing mental well-being than performing such acts for oneself, or keeping track of daily activities (Nelson et al., 2016). These findings indicate that self-focused kindness might be less beneficial for one's mental health compared to practicing other-focused kindness, although studies regarding direct comparison of kindness for the self and others are scarce.

To best understand the potential benefits of other-focused kindness and self-focused kindness across a range of contexts, more work is needed focusing on participants from a range of backgrounds and on a range of mental health outcomes over longer periods of time (Curry et al., 2018). We answer this call in the current study by investigating the effects of kindness in a sample of adults who might be at risk for future mental illness, across a range of mental health outcomes. In addition, we measure the effects of practicing kindness up to 6 months following the kindness intervention.

### Present Research

The purpose of the current study was to examine the efficacy of other-focused kindness with and without reflection and self-focused kindness on mental well-being (including emotional, social, and psychological well-being), depressive symptoms, anxiety, and perceived stress relative to waitlist control. We expected that mental health would improve significantly more when participants reflected on their other-focused kindness compared to those only performing other-focused kindness. We also expected that performing kindness for others with and without reflection would outperform the other two conditions on all of the outcomes, of which more pronounced effects were expected in comparison with waitlist control than in comparison with self-focused kindness.

## Methods

### Design

A parallel randomized controlled trial about the efficacy of different exercises to improve people's well-being was approved by the Ethics Committee of the University of Twente (BCE17240) and registered in the Dutch Trial Register (NTR6786). The current paper describes results according to the CONSORT guidelines (Moher et al., 2010) from four of the five conditions^1^, with an allocation ratio of 1:1:1:1.

### Participants and Procedure

**Figure 1:**
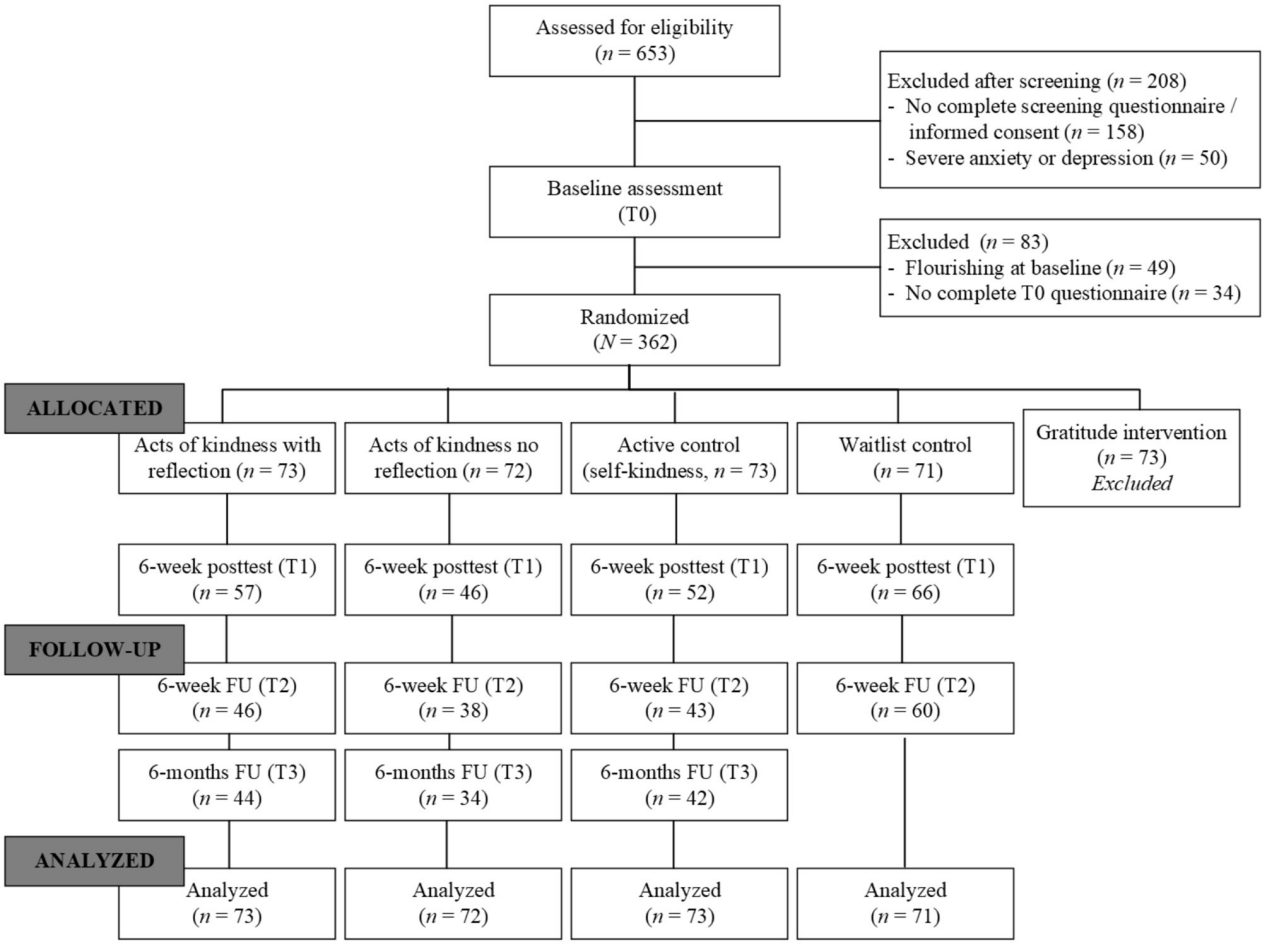
CONSORT flow-chart of participants.

Participants for the complete study^1^ were recruited from the general Dutch population by advertisements at Facebook/LinkedIn (*n* = 425), in a popular psychology magazine (*n* = 156), and in national and regional newspapers (*n* = 72). Participants were required to have sufficient Internet connection and a valid email address, as well as mastery of the Dutch language. Participants who completed the online informed consent procedure were automatically redirected to an online screening questionnaire. Eligible participants were at risk for future mental illness (i.e., low and moderate levels of well-being), but they had no severe complaints which could indicate a clinical disorder. Therefore, we excluded (1) participants who were already flourishing as measured with the Mental Health Continuum-Short Form and by using Keyes' classification guidelines of scoring a 4 or 5 on at least one emotional well-being item together with a score of a 4 or 5 on at least 6 of the 11 social and psychological well-being items (Keyes et al., 2008; Lamers et al., 2011), and (2) participants with severe depressive or anxiety symptoms, indicated by a score >34 on the Center for Epidemiological Studies Depression (CES-D) questionnaire (Radloff, 1977; Bouma et al., 1995; Santor et al., 1995) and a score > 15 on the Generalized Anxiety Disorder-7 (GAD-7) questionnaire (Spitzer et al., 2006; Donker et al., 2009). See Figure 1 for the flow of participants throughout the study.

The final sample for the current study consisted of 289 participants. Participants' ages ranged between 18 and 70 years (*M* = 48.8; *SD* = 9.6). The majority were women (89.6%), married (48.1%), in paid employment (75.4%), living with others (72.0%), and of Dutch nationality (86.2%). Also, the majority had at least a college degree (78.5%).

Prior to data collection, we conducted a power analysis, which showed that 78 participants per condition were needed to obtain a minimal effect size of *d* = 0.45 (Alden and Trew, 2013; Kerr et al., 2015) assuming a two-tailed test with alpha of 0.05 and a power of 0.80 (1 – β). Randomization was stratified by gender and education and conducted using randomizer.org all at once after the deadline for completing the baseline assessment. Participants were not blind for their own condition, but they were not aware of the number and content of the other conditions.

### Conditions

Participants were randomly assigned to one of four conditions: to perform five acts of kindness for others on 1 day per week followed by reflection on those actions on the following day (i.e., *other-focused kindness with reflection; n* = 73), to perform five acts of kindness for others on 1 day per week (i.e., *other-focused kindness without reflection; n* = 72), to perform five acts of kindness for themselves on 1 day per week (i.e., *self-focused kindness; n* = 73), or only to complete all well-being measures and choose a happiness activity after the 6-week follow-up (i.e., *waitlist control, n* = 71; see Supplementary Materials for complete instructions for each condition and the level of adherence per week)^2^. We instructed participants to perform five kind acts on 1 day per week based on prior research indicating that performing five acts of kindness in 1 day led to greater well-being improvements than performing five acts of kindness throughout the week (Lyubomirsky et al., 2005). Participants in the waitlist control group waited for 12 weeks before they chose which activity to improve their well-being they would like to perform. We did not want to wait longer because of the high drop-out risk and for ethical reasons as participants had signed up for receiving a happiness exercise.

As a manipulation check, the day after their kindness day, participants were instructed to list their actions relevant to their assigned conditions. Participants in the *other-kindness with reflection* were also asked to write briefly about their experiences performing the activity, including how they felt, who they were with, and what the activity meant to them and the recipient of their kindness. Participants performed their activities weekly for 6 weeks and completed online assessments at baseline (T0), post-test (T1), 6-week follow-up (T2), and 6-month follow-up (T3). Initially, we planned to do the follow-ups at 6 months and 12 months, but we did not want to risk losing too many participants in the waitlist control condition and decided to bring the dates forward.

### Primary Outcome

#### Mental Well-Being

Participants completed the 14-item Mental Health Continuum-Short Form (MHC-SF) to measure overall mental well-being, including the three-item emotional well-being subscale (e.g., “How often did you feel happy?”), the five-item social well-being subscale (e.g., “How often did you feel that you belonged to a community/social group?”), and the six-item psychological well-being subscale (e.g., “How often did you feel that you liked most aspects of your personality?”; Keyes et al., 2008; Lamers et al., 2011). Each item was rated on a scale from 0 (*never*) to 5 (*every day*). Higher average sores indicate higher levels of mental well-being over the past 4 weeks. Reliability of the total scale ranged between 0.84 and 0.91 for each measurement time point, and between 0.60 and 0.87 for the subscales.

### Secondary Outcomes

#### Depressive Symptoms

Participants completed the 20-item CES-D at screening (1 week before baseline), post-test and at follow-up to measure depressive symptoms during the last week (Radloff, 1977; Bouma et al., 1995). Participants reported their symptoms of depression (e.g., “I felt lonely”) on a scale ranging from 0 (*rarely or none of the time*, <*1 day*) to 3 (*most or all of the time, 5–7 days*). Responses to all items were summed to create a score ranging from 0 to 60. Higher scores indicate greater symptoms of depression. The reliability was good, ranging between 0.84 and 0.93 between time points.

#### Anxiety

The GAD-7 consists of seven items that measure anxiety symptoms during the past 2 weeks (Spitzer et al., 2006; Donker et al., 2009). Participants reported at screening, post-test and follow-up how often they were bothered by several problems (e.g., “not being able to stop or control worrying”) on a scale ranging from 0 (*not at all)* to 3 (*nearly every day*). Responses were summed such that higher total scores (0–21) indicate more symptoms of generalized anxiety. Cronbach's α'*s* showed that the reliability of the scale was acceptable to good, with alpha's ranging from 0.74 to 0.88.

#### Perceived Stress

The 10-item Perceived Stress Scale (PSS) assesses how often a person felt stressed during the past month (Cohen et al., 1994). Participants indicated at baseline, post-test, and follow-up their feelings of stress for each item (e.g., “In the last month, how often have you felt nervous and ‘stressed'?”) on a scale ranging from 0 (*never*) to 4 (*very often*). Responses were summed such that higher scores (0–40) indicate higher levels of perceived stress. The PSS showed good reliability in the present study, with alpha's ranging between 0.85 and 0.89 between time points.

### Process Outcomes

#### Expectations and Motivation

At baseline and after 1 week when completing the first online diary, expectations and motivations were checked through self-developed items. The item about expectations differed slightly between assessments: (1) “How convinced are you about the utility of performing happiness exercises for your sustainable happiness and well-being?” (2) “You now know which happiness exercise you are going to perform. How convinced are you about the utility of performing this happiness exercise for your sustainable happiness and well-being?” Furthermore, motivation was measured with the item: “How motivated are you for doing the/this weekly happiness exercise during 6 weeks?” All these items were scored on a continuous scale from 1 to 10, of which 10 is indicating the highest level of expectations and motivation respectively.

#### Client Satisfaction and Time Spent

At post-test, the participants who were in one of the kindness conditions also completed the 8-item Client Satisfaction Questionnaire-short form (CSQ-8) to assess the level of client satisfaction (Attkisson and Zwick, 1982). Each item has an answer scale from 1 to 4, but the labels differ per item. Five items were recoded as such that a higher sum score (8–32) indicated higher satisfaction with the exercise. The Cronbach's α showed excellent reliability in this study (0.91). An additional question asked participants how much time they had spent on doing the exercise each week, on average (1 = *0–30 min per week*, 2 = *30–60 min per week*, 3 = *more than 2 h per week*).

### Statistical Analyses

All analyses were performed with SPSS version 26.0, using two-tailed tests and *p* < 0.05. Descriptive statistics of demographic variables and outcome measures at baseline, of drop-out and completers, and of the process measures during and after the intervention were calculated and compared between conditions using χ^2^-tests and univariate analysis of variance (ANOVA). Drop-out was defined as participants who completed only the baseline assessment. In addition, the weekly happiness diaries were consulted to obtain the number and content of kind acts for others or themselves that the participants reported on a scale from 0 (*no kind acts*) to 5 (*five kind acts*).

All randomized individuals were analyzed in the allocated intervention arm regardless of whether they had followed the intervention instructions consequently (intention-to-treat principle). In addition, all randomized participants were invited to complete each survey at each time-point although they might have not completed a prior survey. We examined changes in mental well-being and all secondary outcomes over time using multilevel growth curve modeling in *R* (version 0.99.902, NLME package) to account for repeated measures nested within individuals (Singer and Willett, 2003). Within these analyses, we used maximum likelihood estimation based on the available data of the same and other participants.

Because participants were no longer instructed to engage in acts of kindness after 6 weeks, we hypothesized that changes in well-being would be non-linear. Thus, we specified linear and nonlinear changes in well-being. We began with an unconditional growth curve model, specifying linear and quadratic changes over time, which we then compared with hypothesis-testing models.

Composite model: *Y*_*ij*_ = γ_00_ + γ_10_Time_ij_ + γ_20_Time^2^_*ij*_ (ε_ij_ + ζ_oi_ + ζ_1i_Time_ij_ + ζ_2i_Time^2^_*ij*_)
Level 1 model: Y_*ij*_ = π_0i_ + π_1i_Time_*ij*_ + π_2i_Time^2^_*ij*_ + ε_*ij*_
Level 2 models: π_0i_ = γ_00_ + ζ_0i_, π_1i_ = γ_10_ + ζ_1i_, and π_2i_ = γ_20_ + ζ_2i_

After the 6-week follow-up, participants in the waitlist condition started with the self-chosen well-being activity. Therefore, we present two sets of analyses: one comparing the effects of kindness with waitlist control from baseline to the 6-week follow-up and a second analysis comparing the three kindness conditions from baseline to the 6-month follow-up. Time was centered on the second time point (post-test). In hypothesis-testing models, each condition was dummy-coded (waitlist control or self-kindness as reference group) and entered as between-subjects predictors in the second level models.

## Results

### Baseline Characteristics and Drop-Out

The four conditions did not differ on most of the characteristics mentioned in Table 1 (χ^2^s < 6.02*, p*s > 0.111) and on the outcome measures at baseline (see Table 2; *F*s < 2.36, *p*s > 0.072). However, participants in the *waitlist control* were more often of Dutch nationality, χ^2^(3) = 8.21, *p* = 0.042, and less often living with children, χ^2^(3) = 8.17, *p* = 0.043, compared to the other three groups.

**Table 1:** Baseline characteristics of the participants performing kind acts for others or themselves, or who were on the waitlist.

	**Other-focused kindness with reflection** **(*n* = 73)**	**Other-focused kindness without reflection** **(*n* = 72)**	**Self-focused kindness** **(*n* = 73)**	**Waitlist control** **(*n* = 71)**
Age, *M (SD)*	48.0 (9.0)	48.6 (10.4)	48.4 (9.2)	48.8 (9.6)
Female gender, *n* (%)	65 (89.0)	64 (88.9)	66 (90.4)	64 (90.1)
Education, *n* (%)				
Low	3 (4.1)	1 (1.4)	3 (4.1)	3 (4.2)
Intermediate	13 (17.8)	14 (19.4)	13 (17.8)	12 (16.9)
High	57 (78.1)	57 (79.2)	57 (78.1)	56 (78.9)
Marital status, *n* (%)				
Married	33 (45.2)	37 (51.4)	34 (46.6)	35 (49.3)
Divorced or widowed	16 (22.5)	19 (26.8)	18 (25.4)	18 (25.4)
Never been married	24 (32.9)	16 (22.2)	21 (28.8)	18 (25.4)
Dutch nationality, *n* (%)	59 (80.8)	62 (86.1)	60 (82.2)	68 (95.8)
Living alone, *n* (%)	19 (26.0)	19 (26.4)	23 (31.5)	20 (28.2)
Living with children, *n* (%)	42 (57.5)	43 (59.7)	38 (52.1)	27 (38.0)
Paid employment, *n* (%)	60 (82.2)	49 (68.1)	59 (80.8)	50 (70.4)

**Table 2:** Means (SDs) for mental health outcomes on each assessment by condition.

	**Other-focused kindness with reflection** **(*n* = 73)**	**Other-focused kindness without reflection** **(*n* = 72)**	**Self-focused kindness** **(*n* = 73)**	**Waitlist control** **(*n* = 71)**
**Mental well-being**				
Pre-test	2.48 (0.55)	2.71 (0.43)	2.58 (0.54)	2.60 (0.54)
Post-test	2.86 (0.62)	2.99 (0.61)	2.87 (0.71)	2.73 (0.66)
6-week follow-up	2.89 (0.64)	3.00 (0.55)	2.84 (0.61)	2.72 (0.62)
6-month follow-up	2.84 (0.69)	3.10 (0.59)	3.03 (0.72)	-
**Emotional well-being**				
Pre-test	2.60 (0.69)	2.82 (0.59)	2.76 (0.72)	2.74 (0.71)
Post-test	2.98 (0.66)	3.21 (0.73)	3.05 (0.84)	2.88 (0.79)
6-week follow-up	2.99 (0.78)	3.04 (0.68)	2.98 (0.68)	2.81 (0.82)
6-month follow-up	3.05 (0.70)	3.35 (0.72)	3.20 (0.80)	–
**Social well-being**				
Pre-test	2.34 (0.65)	2.56 (0.55)	2.39 (0.58)	2.39 (0.62)
Post-test	2.66 (0.72)	2.70 (0.66)	2.65 (0.69)	2.48 (0.69)
6-week follow-up	2.65 (0.71)	2.81 (0.63)	2.60 (0.65)	2.55 (0.63)
6-month follow-up	2.57 (0.73)	2.86 (0.56)	2.81 (0.68)	–
**Psychological well-being**				
Pre-test	2.54 (0.61)	2.77 (0.51)	2.65 (0.61)	2.70 (0.60)
Post-test	2.96 (0.73)	3.13 (0.72)	2.96 (0.82)	2.87 (0.74)
6-week follow-up	3.04 (0.74)	3.13 (0.67)	2.97 (0.67)	2.82 (0.71)
6-month follow-up	2.95 (0.85)	3.18 (0.79)	3.15 (0.88)	–
**Depressive symptoms**				
Pre-test	21.03 (6.75)	19.40 (7.88)	18.90 (7.23)	19.44 (7.71)
Post-test	16.56 (10.01)	14.26 (6.99)	15.08 (10.20)	17.78 (9.93)
6-week follow-up	17.50 (11.46)	14.18 (8.32)	13.95 (6.83)	17.69 (10.96)
6-month follow-up	17.19 (11.82)	13.47 (8.47)	15.10 (11.05)	–
**Anxiety**				
Pre-test	6.66 (3.28)	6.47 (3.46)	5.78 (2.85)	6.31 (2.72)
Post-test	5.72 (4.28)	4.93 (3.57)	4.76 (3.90)	6.73 (4.45)
6-week follow-up	5.21 (4.32)	5.16 (3.98)	4.79 (2.68)	6.39 (5.03)
6-month follow-up	6.24 (4.86)	4.74 (3.19)	4.92 (4.27)	–
**Perceived stress**				
Pre-test	20.32 (5.90)	19.32 (5.84)	18.41 (5.39)	19.08 (5.34)
Post-test	16.97 (6.70)	14.41 (5.59)	15.20 (5.79)	16.64 (6.16)
6-week follow-up	16.66 (6.81)	14.65 (5.11)	15.56 (4.91)	16.71 (6.24)
6-month follow-up	16.76 (7.16)	14.85 (5.62)	14.60 (6.57)	–

Of the 289 participants who began the study, 58 participants (20.1%) did not complete any of the post-test or follow-up assessments. Participants in the three kindness conditions were significantly more likely to drop out compared to waitlist control (*other-kindness with reflection* = 16.4%, *other-kindness without reflection* = 30.6%, *self-kindness* = 27.4%, *waitlist* = 5.6%), χ^2^(3) = 17.20, *p* = 0.001. In addition, drop-outs were significantly younger (*M* = 43.3, *SD* = 10.5) than participants who completed at least two assessments (*M* = 50.1, *SD* = 8.9), *F*(287) = 25.60, *p* < 0.001. All 289 participants who completed at least the baseline survey were included in analyses.

### Changes in Mental Well-Being

**Figure 2:**
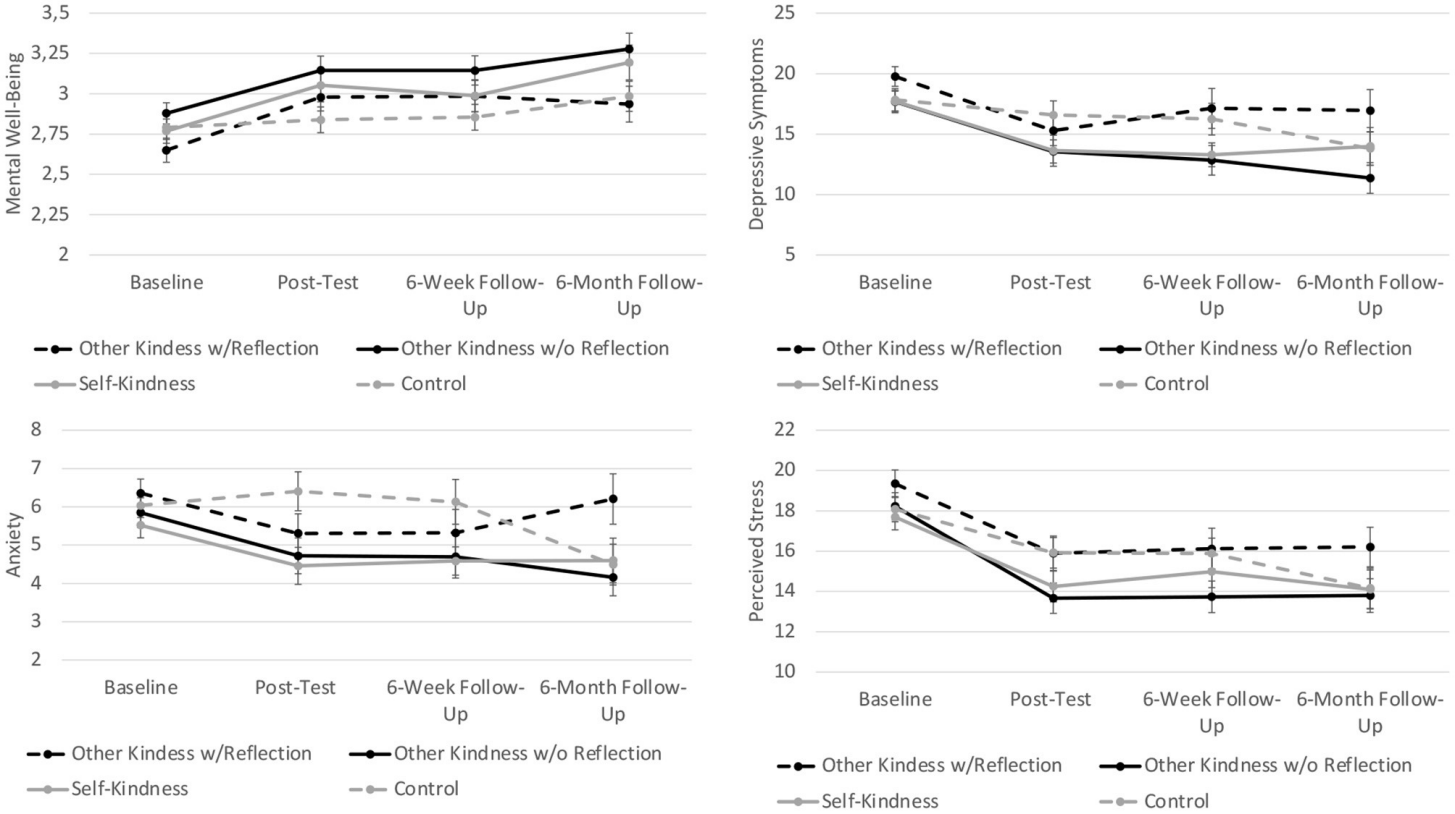
Average changes in well-being and psychological distress outcomes by condition.

Relative to waitlist control, *other-focused kindness with and without reflection* led to significant linear improvements in mental well-being up to 6-week follow-up (γs > 0.10, *p*s < 0.047, *d*^3^ = 0.38 and 0.42, respectively). *Self-focused kindness* did not lead to any changes in mental well-being compared to waitlist control, and the trajectories of the kindness conditions did also not differ from each other (γs <-0.12, *p*s >0.111). In addition, the three kindness conditions did not differ from each other in linear or non-linear changes in mental well-being up to 6-months follow-up (γ*s* < −0.04*, p*s >0.332). See Tables 3, 4 for parameter estimates and model fit indices and Figure 2 for a visual presentation of the trajectories.

**Table 3:** Parameter estimates and goodness of fit indices for multilevel growth curve models predicting changes in mental well-being up to 6-weeks follow-up comparing other-focused kindness and self-focused kindness with waitlist control.

		**Mental well-being**			
**Fixed effects**	**Parameter**	**Model 1: Unconditional quadratic growth**	**Model 2: Other-focused kindness w/reflection vs. other-focused kindness w/o reflection**	**Model 3: Other-focused kindness vs. self-focused**	**Model 4: Kindness vs. waitlist**
**Status at post-test, π_oi_**					
Intercept	γ_00_	2.84^***^ (0.04)	3.00^***^ (0.09)	2.85^***^ (0.09)	2.71^***^ (0.08)
Other-focused kindness w/reflection	γ_01_		−0.17 (0.12)	−0.02 (0.12)	0.12 (0.12)
Other-focused kindness w/o reflection	γ_02_			0.15 (0.12)	0.28^*^ (0.12)
Self-focused kindness	γ_03_				0.14 (0.12)
**Linear rate of change, π_1i_**					
Time	γ_10_	0.15^***^ (0.02)	0.18^***^ (0.04)	0.16^***^ (0.04)	0.08^*^ (0.03)
Other-focused kindness w/reflection	γ_11_		0.01 (0.05)	0.03 (0.05)	0.11^*^ (0.05)
Other-focused kindness w/o reflection	γ_12_			0.02 (0.05)	0.10^*^ (0.05)
Self-focused kindness	γ_13_				0.08 (0.05)
**Quadratic rate of change, π_2i_**					
Time^2^	γ_20_	−0.10^**^ (0.03)	−0.11 (0.07)	−0.11^†^ (0.06)	−0.03 (0.05)
Other-focused kindness w/reflection	γ_21_		−0.05 (0.09)	−0.05 (0.09)	−0.12 (0.08)
Other-focused kindness w/o reflection	γ_22_			0.00 (0.09)	−0.07 (0.09)
Self-focused kindness	γ_23_				−0.08 (0.08)
**Goodness-of-fit**					
Deviance		980.25	964.15	964.15	964.15
Δχ^2^			16.09^†^	16.09^†^	16.09^†^

† *p < 0.10,*

* *p < 0.05,*

** *p < 0.01,*

*** *p < 0.001*.

**Table 4:** Parameter estimates and goodness of fit indices for multilevel growth curve models predicting changes in mental well-being up to 6-months follow-up comparing other-focused kindness with self-focused kindness.

		**Mental well-being**		
**Fixed effects**	**Parameter**	**Model 1: Unconditional quadratic growth**	**Model 2: Other-focused kindness w/reflection vs. other-focused kindness w/o reflection**	**Model 3: Other-focused kindness vs. self-focused**
**Status at post-test, π_oi_**				
Intercept	γ_00_	2.86^***^ (0.04)	2.97^***^ (0.08)	2.82^***^ (0.08)
Other-focused kindness w/reflection	γ_01_		−0.18^†^ (0.10)	−0.02 (0.10)
Other-focused kindness w/o reflection	γ_02_			0.16 (0.11)
**Linear rate of change, π_1i_**				
Time	γ_10_	0.20^***^ (0.02)	0.20^***^ (0.04)	0.18^***^ (0.04)
Other-focused kindness w/reflection	γ_11_		0.02 (0.05)	0.04 (0.05)
Other-focused kindness w/o reflection	γ_12_			0.02 (0.06)
**Quadratic rate of change, π_2i_**				
Time^2^	γ_20_	−0.07^***^ (0.02)	−0.06^*^ (0.03)	−0.05^†^ (0.03)
Other-focused kindness w/reflection	γ_21_		−0.02 (0.04)	−0.04 (0.04)
Other-focused kindness w/o reflection	γ_22_			−0.01 (0.04)
**Goodness-of-fit**				
Deviance		903.53	920.80	920.80
Δχ^2^			17.27^**^	17.27^**^

† *p < 0.10,*

* *p < 0.05,*

** *p < 0.01,*

*** *p < 0.001*.

### Changes in Psychological Distress

Relative to waitlist control, *other-focused kindness without reflection* led to significant linear improvements through the 6-week follow-up in depressive symptoms and perceived stress (γs >-1.31, *p*s < 0.035, *d* = 0.55 and 0.54, respectively) and to quadratic changes in anxiety, γ_22_ = 1.29, *SE* = 0.65, *t*(394) = 1.97, *p* = 0.050. Furthermore, *other-focused kindness with reflection* and *other-focused kindness without reflection* did not differ from each other in linear or non-linear trajectories in any outcome. Relative to *self-focused kindness, other-focused kindness without reflection* led to significant linear improvements in perceived stress up to 6-week follow-up, γ_12_ = −1.31, *SE* = 0.57, *t*(393) = −2.30, *p* = 0.022, *d* = 0.47.

Up to 6-months follow-up, most comparisons between the three kindness conditions did not significantly differ from each other. However, *other-focused kindness without reflection* led to significant linear improvements in perceived stress relative to *self-focused kindness*, γ_12_ = −1.26, *SE* = 0.59, *t*(388) = −2.12, *p* = 0.034, *d* = 0.67.

### Expectations, Motivation, and Client Satisfaction

Participants in the three kindness conditions did not significantly differ regarding how convinced they were about the utility of doing happiness exercises at baseline (*M* = 7.15, *SD* = 1.38), *F*(215) = 1.53, *p* = 0.219, and after 1 week practicing (*M* = 6.32, *SD* = 1.79), *F*(192) = 0.02, *p* = 0.983. Participants were also highly motivated to practice a happiness exercise at baseline (*M* = 8.05, *SD* = 1.26), *F*(215) = 0.34, *p* =0.812, and after 1 week practicing (*M* = 7.28, *SD* = 1.81), *F*(192) = 1.30, *p* = 0.274, and highly interested in positive psychology (*M* = 8.48, *SD* = 1.13), *F*(215) = 0.81, *p* = 0.445, which all did not differ between conditions.

The level of satisfaction with the kindness exercises was moderate for each condition (*other-kindness with reflection M* = 19.4, *SD* = 5.06; *other-kindness without reflection M* = 19.9, *SD* = 3.89, *self-kindness M* = 19.8, *SD* = 4.80), *F*(153) = 0.20, *p* = 0.817. However, participants practicing *other-focused kindness* mainly spent 0–30 min per week on the intervention (*other-kindness with reflection* = 64.7%, *other-kindness without reflection* = 70.7%, *self-kindness* = 44.4%), while *self-focused kindness* mainly spent 30–60 min per week (*other-kindness* with reflection = 19.6%, *other-kindness without reflection* = 14.6%, *self-kindness* = 37.8%), χ^2^(4) = 8.38, *p* = 0.079.

### Performed Activities

Participants in the two *other-focused kindness* conditions performed 2,126 kind acts in total (*other-kindness with reflection M* = 15.7, *SD* = 7.87; *other-kindness without reflection M* = 16.8, *SD* = 8.68), *F*(130) = 0.50, *p* = 0.479. These kind acts could be mainly categorized in moral support (e.g., investing time, comforting, showing interest, greeting strangers; complementing, expressing gratitude) and physical support (e.g., helping, serving, doing chores, volunteering), but also donating material goods or money to others were mentioned (see Supplementary Materials for some specific examples). Participants in the *self-focused kindness* condition performed 1,143 kind acts (*M* = 16.8, *SD* = 8.61), *F*(198) = 0.35, *p* = 0.704, which could be mainly categorized in self-indulgent behavior (e.g., indulging in food or drinks, lazy leisure, material goods, wellness). In addition, some of these self-indulgent behaviors also involved other people (e.g., “watching a movie with my partner,” “having a nice lunch with a former colleague”; “went shopping with a friend”). A minority of the kind acts were mainly or partly about mindfulness and self-compassion, cherish meaningful relationships with others, and active leisure such as physical activity and hobbies.

Participants in the *waitlist condition* were provided instructions after the 6-week follow-up about the kindness and gratitude conditions and given the opportunity to choose an activity to improve their well-being. Of the 60 participants who completed the 6-weeks follow-up, 66.7% chose to perform the *gratitude intervention*, 25.0% chose to perform *self-focused kindness*, and 6.7% chose *other-focused kindness*. One person did not want to do an exercise (1.7%).

## Discussion

This randomized controlled trial demonstrated that other-focused kindness with and without reflection did not differ from each other on any of the outcome measures. Other-focused kindness with and without reflection were more effective in improving mental well-being up to 6-weeks follow-up in a sample at risk for future mental illness than waitlist control, but not up to 6-months follow-up. In addition, significant effects were found for other-focused kindness without reflection on depression, anxiety and perceived stress relative to waitlist control up to 6-weeks follow-up. Self-focused kindness fell usually somewhere between the other-focused kindness and waitlist control conditions, showing mainly no significant differences relative to these conditions. However, significant changes on perceived stress up to 6-months follow-up were found for other-focused kindness without reflection relative to self-focused kindness.

To our knowledge, the current study was the first to compare other-focused kindness with and without reflection. Contrary to expectations, adding reflection to other-focused kindness did not lead to greater improvements in any of the outcomes. In fact, kindness without reflection outperformed the other conditions on psychological distress when comparing each condition to waitlist control; however, no significant differences were found between the two other-focused kindness conditions. A possible explanation for not finding additional effects of reflection might be that the reflective questions were too brief and that adding an expressive writing exercise for at least 15 min on one or more days about their practiced kindness could have been more effective (Toepfer et al., 2016; Aknin et al., 2020).

Another possibility is that the instructions of performing five kind acts on 1 day was too difficult as also indicated by low adherence levels per week. The weekly reflections could have shifted from its content and possible benefits for themselves and others to reflections about the difficulty and repetitive character of the task. More variation is argued to be one of the factors for more lasting effects of positive interventions (Lyubomirsky and Layous, 2013). In addition, how participants reflect on their kind acts might diminish the benefits of reflection. For example, the reflection task might have led to critical reflections in which participants felt ambiguous by the fact that they felt good by doing good for others and might believe they were doing the acts too much for their own benefits instead of altruistically help others. Alternatively, to the extent that reflection led participants to explain or over-analyze their positive experiences, they may no longer reap the benefits of those actions (e.g., Lyubomirsky et al., 2006; Wilson and Gilbert, 2008).

The current study also adds to the literature that other-focused kindness is beneficial in enhancing overall mental health, as indicated by emotional, social, and psychological well-being and partly by improved levels of depression, anxiety, and perceived stress. To date, most studies about the efficacy of performing prosocial behavior assessed emotional well-being (happiness, life-satisfaction or positive affect; e.g., Ouweneel et al., 2014; Nelson et al., 2015) and sometimes also psychological distress (e.g., Mongrain et al., 2011; Kerr et al., 2015). Our study aligns most strongly with prior findings indicating practicing kindness toward others was more beneficial for one's multidimensional well-being in comparison with tracking daily activities (Nelson et al., 2016). We also demonstrated that kindness for others leads to reductions in depressive symptoms, anxiety and perceived stress, although these results were only significant for the group who did not reflect on their performed kind acts.

Importantly, the effects of the three kindness conditions mainly followed a nonlinear trajectory up to 6 months follow-up. These findings indicate that practicing kindness for others or for the self are both successful strategies for improving one's mental health, but that they may not lead to sustained well-being without continued practice. As indicated by our significant non-linear trajectories, most participants who practiced kindness for others or oneself demonstrated an improvement in well-being up to 6 weeks following the intervention, followed by a subsequent decline in well-being by 6 months follow-up. These findings also show that it is important for future research to follow participants for a longer time period because significant effects might diminish over time for some but not all activities or programs (e.g., Schotanus-Dijkstra et al., 2017a).

A surprising finding compared to prior research (e.g., Nelson et al., 2016) was that other-focused kindness was mainly not more effective relative to self-focused kindness, although self-focused kindness was also not more effective relative to waitlist control. A possible explanation may be that participants reported that they spent more time on their self-focused kind acts than on other-focused kind acts. This might be due to the nature and duration of the performed acts such as a wellness related treatment (self-focused) vs. encouraging a friend over the phone (other-focused). Future research could add a time frame of how long a kind activity for others or the self might last (e.g., between 5 and 15 min). In addition, some activities might be more easy to integrate in daily life and thereby be more effective (e.g., self-focused kindness), while other behaviors might be more effective by putting in greater effort (e.g., other-focused kindness; Schiffer and Roberts, 2018). Furthermore, where positive emotions might be an important underlying mechanism of positive psychological interventions in general and of acts of kindness in particular (Nelson et al., 2016; Hendriks et al., 2020), the type of emotions that influence well-being might differ per kindness type. For example, self-focused kindness might elicit low-arousal positive emotions such as relaxation and satisfaction, while other-focused kindness might elicit high-arousal positive emotions such as joy, happiness, and surprise.

Interestingly, other-focused kindness without reflection was more effective in relieving perceived stress compared to self-focused kindness. When people perceive stress, a common strategy is to be kind to yourself by seeking relaxation and self-indulgent behavior. However, the current study suggests that helping others might be a more sufficient strategy for relieving perceived stress. Focusing on others might offer distraction from one's perceived stress and may provide opportunities to put things in perspective, which might result in lowered feelings of stress. By contrast, prior studies have indicated that the level of self-compassion is more predictive for students' well-being than their levels of stress or social support (Neely et al., 2009), and research also found that self-compassionate people can better cope with daily stresses and chronic illnesses (Sirois et al., 2015). This discrepancy with our findings could be explained by the fact that self-compassion also involves common humanity and mindfulness and defines self-kindness more in terms of being less self-critical (Neff, 2003). The self-kindness activities conducted in the current study seem different from those encouraged by self-compassion research. Although our study indicates that other-focused and self-focused kindness can be beneficial for your mental health, when feeling stressed, it might pay off to help others first before you indulge yourself. Yet, this implication should be tested directly in a sample with elevated levels of (manipulated) stress, especially because we did not find an effect on perceived stress for other-focused kindness with reflection.

## Limitations

We expand prior evidence on the efficacy of performing acts of kindness by examining the value of adding reflection to the intervention and by comparing this positive psychology intervention with both a self-focused kindness condition and a waitlist control group. We also selected participants with low or moderate levels of well-being who manifested no severe clinical complaints and followed participants for a longer time span. However, some limitations also apply.

First, although we conducted the power analysis in line with prior kindness studies, the meta-analysis of Curry et al. (2018) suggest that a sample size of at least 202 per cell is needed. We might have more power than prior studies using student samples because our more vulnerable participants had more room to grow; however, future studies should pay attention to optimize power. Second, full adherence to the program gradually decreased from approximately 70 to 35% over the 6-week intervention period with participants in the other-focused kindness without reflection condition demonstrating the greatest attrition and waitlist participants demonstrating the lowest rates of attrition. Although all participants were included in our analyses, differential drop-out rates across conditions may have influenced our results. Third, although the use of a no-treatment control group gives more power, it also limits strong evidence for the efficacy of practicing kindness because of differences between groups attributed to differences in effort, motivation, receiving attention, or other factors unrelated to kindness behaviors. Fourth, the self-selected sample consisted mainly of higher-educated native Dutch females which is consistent with many prior positive intervention studies (e.g., Mongrain et al., 2011; Schotanus-Dijkstra et al., 2017a), but with the consequence that the results are not directly applicable to the general population.

## Concluding Remarks

In sum, our study demonstrated that focusing attention on others via acts of kindness not only improves positive psychological well-being, but also reduces perceived stress and symptoms of depression and anxiety in a sample of individuals vulnerable to mental illness. Positive psychologists should make greater efforts to recruit and target people with low or moderate well-being and to discover in larger samples for whom and why engaging in small kindnesses for others and the self is a useful strategy to feel better. In addition, researchers could apply the current methodology to other positive psychological interventions by focusing not only on student or clinical samples, but on those at risk for future mental illness as well.

## Data Availability Statement

The raw data supporting the conclusions of this article will be made available by the authors, without undue reservation.

## Ethics Statement

The studies involving human participants were reviewed and approved by the Ethics Committee of the University of Twente (BCE17240). The participants provided their written informed consent to participate in this study.

## Author Contributions

ETB and MSD contributed to conception and design of the study. SKNC and MSD performed the statistical analyses and wrote the first draft of the manuscript. All authors contributed to manuscript revision, read, and approved the submitted version.

## Conflict of Interest

The authors declare that the research was conducted in the absence of any commercial or financial relationships that could be construed as a potential conflict of interest.

## Publisher's Note

All claims expressed in this article are solely those of the authors and do not necessarily represent those of their affiliated organizations, or those of the publisher, the editors and the reviewers. Any product that may be evaluated in this article, or claim that may be made by its manufacturer, is not guaranteed or endorsed by the publisher.

## Footnotes

^1^A fifth condition, unrelated to the kindness conditions, was run simultaneously for comparison with waitlist control of which the results are described elsewhere (Bohlmeijer et al., 2020). The fifth condition consisted of a 6-week gratitude intervention in which six different exercises were used, while the current study focused on different kindness conditions in which the same activity was used each week, during 6 weeks. The distinct study aims were reported before the start of the study in the Dutch Trial Register (NTR6786). We report this fifth condition as a footnote for the sake of clarity.

^2^Given that our power analysis indicated we would need 78 participants per condition, we opted to focus our study on the primary conditions of interest (other-focused kindness with and without reflection) to maximize power rather than to fully cross reflection with the focus of kindness.

^3^Effect size *d* was calculated with this equation: γ ^*^(duration – 1)/*SD*_*raw*_ (Feingold, 2009, 2015). This effect size estimate is only appropriate for linear slopes and represents an estimate of the standardized mean difference (Cohen's *d*) between groups at the end of the study.
