# Supplementary material for: Practicing Other-Focused Kindness and Self-Focused Kindness Among Those at Risk for Mental Illness: Results of a Randomized Controlled Trial
Source: Front Psychol. 2021 Oct 14;12:741546. doi: 10.3389/fpsyg.2021.741546 (PMC8551549; doi:10.3389/fpsyg.2021.741546)
Supplement: Supplementary file 1 [file Table_1.DOCX]

**Supplemental Material**

**Instructions for Participants**

***Other-Focused Kindness***

In our daily lives, we all perform acts of kindness—both large and small—for others. Examples include cooking dinner for friends or family, doing a chore for a family member or neighbor, holding the door for someone, paying for someone’s coffee in line behind you, visiting an elderly relative or volunteering for a local organization.

Within the next five days, you pick one day as your kindness day. On this day, you are to perform *five* nice things for others, *all five in one day*. These acts of kindness do not need to be for the same person, the person may or may not be aware of the act, and the act may or may not be similar to the acts listed above. *The day after your kindness day*, we ask you to report in your happiness diary (via the link below) what nice things you chose to perform. Please do not perform any kind acts that may place yourself or others in danger.

***Self-Focused Kindness***

In our daily lives, we all perform acts of kindness for others, but we often neglect to do nice things for ourselves – small and simple things that require relatively little extra money or effort. Examples include treating yourself on a special coffee or on your favorite pastry, magazine or meal, taking a 5-minute break from work or study, give yourself a compliment or go for a walk. Now and then, you can treat yourself on something bigger, such as a massage or spending time on your favorite hobby.

Within the next five days, you pick one day as your kindness day. On this day, you are to perform *five* nice things for yourself, *all five in one day*. These acts of kindness should be something out of the ordinary that you do for yourself with a little extra effort and may or may not be similar to the acts listed above. *The day after your kindness day*, we ask you to report in your happiness diary (via the link below) what nice things you chose to perform. Please do not perform any kind acts that may place yourself or others in danger.

***Waitlist Control***

You are allocated to the group who can choose which of the three happiness exercises you would like to perform. The so called “flexible group”. You can choose the activity that fits best to your needs to improve your happiness and well-being. However, before you can choose this activity, we would like to monitor your normal fluctuations in your level of happiness. Therefore, you will complete some questionnaires during the upcoming weeks—the other groups receive the same amount of questionnaires at the same time. Over 12 weeks, you can choose your happiness exercise. You might be disappointed about not starting with the exercise right away. That is understandable. However, we would like to emphasize the importance of this flexible group in our study. We hope you can keep your patience for a while and help us by completing the questionnaires about your happiness and well-being in the meantime.

***Happiness Diary***

Participants in the acts of kindness conditions and the self-kindness condition received a link to their online happiness diary wherein they were asked: (1) how many kind acts they performed yesterday on a 6 point scale (5 to none), and (2) please provide a brief description of each kind act they performed (and who was the recipient – only for the participants in the kindness for others conditions). The only difference between the two acts of kindness conditions was that the group with reflection received an additional question in their diary: “Please write briefly about the meaning of doing these kind acts: How was it for you to do the activity? For example: What did you feel, what did you do, who were you with or who was it for, what did it bring you (and the other)?”

**Table 1**

*Example Responses of the Performed Kind Acts for each Condition*

| *Other-Focused Kindness* | *“Invited a friend to stop by and eat soup”*  *“Encouraged a friend on the phone”*  *“Sent a message to a friend in order to check how she is”*  *“Asked a colleague if I could help with a task”*  *“Called back a man in a restaurant who forgot his shawl”*  *“Made a delicious lunch for someone with a lot of diet wishes”*  *“Ironing for my partner”*  *“Volunteering: Visiting a man with depths”*  *“Donated money to a charity organization”*  *“Smiled to a passer-by”*  *“Thanked the bus driver after a ride”* |
| --- | --- |
| *Self-Focused Kindness* | *“Enjoyed an extra treat: A brownie with my coffee”*  *“Watched an episode of my favorite TV series”*  *“Spend some time on my own this afternoon, simply doing nothing on the couch”*  *“Took a nap”*  *“Bought a book and had it wrapped up as a gift for myself”*  *“Took a long bath”*  *“I used luxury body lotion”*  *“Consciously made time in the morning for some yoga, meditation and enjoyed the shower”*  *“Wrote down what I am grateful for”*  *“Invited a friend for dinner”*  *“Went for a walk after a day of sitting”*  *“Knitted for an hour”*  *“Quality time with the dog”* |

**Table 2**

*Adherence to the activities performed per week*

|  | Other-focused kindness with reflection  (*n* = 73) | Other-focused kindness without reflection  (*n* = 72) | Self-focused kindness  (*n* = 73) |
| --- | --- | --- | --- |
| Week 1 |  |  |  |
| No activities or missing | 9 (12.3%) | 13 (18.1%) | 6 (8.2%) |
| 1-3 activities | 11 (15.1%) | 12 (16.7%) | 20 (23.3%) |
| 4 or 5 activities | 53 (72.6%) | 47 (65.3%) | 50 (68.5%) |
| Week 2 |  |  |  |
| No activities or missing | 23 (31.5%) | 25 (34.7%) | 18 (24.7%) |
| 1-3 activities | 15 (20.5%) | 18 (25.0%) | 19 (26.0%) |
| 4 or 5 activities | 35 (47.9%) | 29 (40.3%) | 36 (49.3%) |
| Week 3 |  |  |  |
| No activities or missing | 30 (41.1%) | 32 (44.4%) | 29 (39.7%) |
| 1-3 activities | 11 (15.1%) | 13 (18.1%) | 12 (16.4%) |
| 4 or 5 activities | 32 (43.8%) | 27 (37.5%) | 32 (43.8%) |
| Week 4 |  |  |  |
| No activities or missing | 34 (46.6%) | 32 (44.4%) | 28 (38.4%) |
| 1-3 activities | 14 (19.2%) | 15 (20.8%) | 14 (19.2%) |
| 4 or 5 activities | 25 (34.2%) | 25 (34.7%) | 31 (42.5%) |
| Week 5 |  |  |  |
| No activities or missing | 40 (54.8%) | 38 (52.8%) | 30 (41.1%) |
| 1-3 activities | 13 (17.8%) | 10 (13.9%) | 20 (27.4%) |
| 4 or 5 activities | 20 (27.4%) | 24 (33.3%) | 23 (31.5%) |
| Week 6 |  |  |  |
| No activities or missing | 29 (39.7%) | 29 (40.3%) | 32 (43.8%) |
| 1-3 activities | 19 (26.0%) | 16 (22.2%) | 19 (26.0%) |
| 4 or 5 activities | 25 (34.2%) | 27 (37.5%) | 22 (30.1%) |
